# Glutamine powder-induced hepatotoxicity: it is time to understand the side effects of sports nutritional supplements 

**Published:** 2020

**Authors:** Behzad Hatami, Ali Saffaei, Faezeh Jamali, Mohammad Abbasinazari

**Affiliations:** 1 *Gastroenterology and Liver Diseases Research Center, Shahid Beheshti University of Medical Sciences, Tehran, Iran *; 2 *Student Research Committee, Department of Clinical Pharmacy, School of Pharmacy, Shahid Beheshti University of Medical Sciences, Tehran, Iran*; 3 *Department of Clinical Pharmacy, School of Pharmacy, Shahid Beheshti University of Medical Sciences, Tehran, Iran *

**Keywords:** Glutamine, Hepatotoxicity, Side effects, Pharmacovigilance, Supplements

## Abstract

Glutamine has been considered as a dietary supplement with a non-essential amino acid structure. Some studies have found that liver failure may be associated with a high plasma glutamine level. Consumption of this product may be linked to potential adverse effects. This report describes the first case of glutamine-induced hepatotoxicity. A 35-year-old female athlete with severe abdominal pain and scleral icterus was referred to the hospital. She had been taking glutamine powder for the past three weeks. Impaired liver function test and imaging evaluation suggested hepatotoxicity. Glutamine consumption was discontinued and the patient was closely monitored. Finally, after two weeks, the patient recovered successfully. This novel case was the first report regarding glutamine-induced hepatotoxicity. Health care providers must know that consumption of dietary supplements such as glutamine may be associated with serious side effects. Liver damage is a possible side effect of glutamine. Hence it is necessary to consider hepatotoxicity as an adverse reaction in case of glutamine supplement consumption.

## Introduction

 The risk of drug-induced liver injury is significantly different given the drug. In the United States and Europe, antimicrobial agents are the main culprit, for example by amoxicillin/clavulanate, while in Asians, dietary and herbal supplements are the major cause of drug-induced liver injury (1). The main hepatotoxic agents include anabolic steroids, green tea extract, and multi-ingredient nutritional supplements. Anabolic steroids advertised as body building supplements characteristically induce a prolonged cholestasis. Green tea extract and other agents, in contrast, lead to an acute-hepatitis-like injury (2). Today, athletes use several strategies to achieve success in their sport competition such as nutritional supplements. These supplements are used as a nutritional supplement and is added to the usual diet mostly including mineral products, vitamins, herbal products, creatine, caffeine, and amino acids (3, 4). Note that despite the great usage of these supplements, the beneficial effects are controversial. Irrational and excessive use of these supplements could increase the adverse effects. Also, there are some concerns over long-term usage of these supplements which can be associated with more severe adverse effects, ranging from simple physical discomfort to life-threating diseases (5, 6). Awareness of these potential life-threating diseases and their symptoms is essential for athletes, coaches, physicians, and other health care providers (7). Herein, a case of severe hepatotoxicity is described following glutamine powder consumption. 

## Case Report

**Figure 1:**
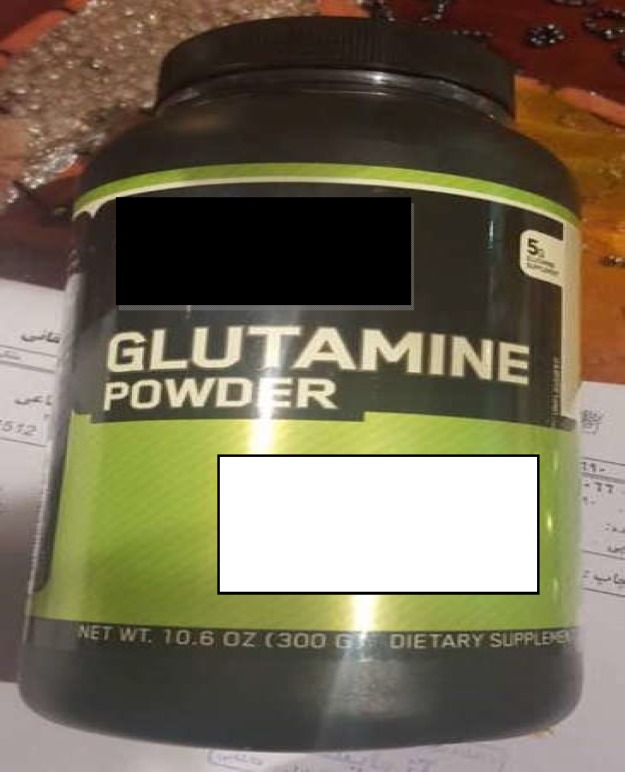
Glutamine powder used by the patient

A 35-year-old female body builder (body mass index: 20.39) was referred to our hospital for evaluation of acute onset right upper quadrant abdominal pain radiating to the shoulders over the last three days. This was associated with lethargy, anorexia, nausea, vomiting, fever, chills, yellowish discoloration of skin and urine darkness for eight days. During the time of admission, she was afebrile and had stable vital signs. Drug history did not show any notable point, except glutamine powder consumption. She was not on a specific diet regimen and she did not take any food supplements. She did not use any recreational drugs either. She was taking glutamine powder (10 g powder/day equal to 170 mg pure glutamine) for the past three weeks based on the advice of her coach (Figure 1). 

The consumed powder only contained glutamine. The patient admitted to occasional and clinically insignificant alcohol consumption with her last intake three months back. She denied any chronic diseases in her past medical history. On the physical examination, scleral icterus and a mild splenomegaly were observed. The laboratory results showed impaired liver function in the tests. Total bilirubin level was 14.8 mg/dL (normal range up to 2 mg/dL), conjugated bilirubin level was 10 mg/dL (normal range less than 1 mg/dL), aspartate transaminase (AST) level was 2500 IU/L (normal range up to 31 IU/L), alanine transaminase (ALT) level was 2400 IU/L (normal range up to 32 IU/L), and alkaline phosphatase (ALP) level was 492 U/L (normal range up to 279 IU/L). The international normalized ratio was 1.4. 

**Figure 2:**
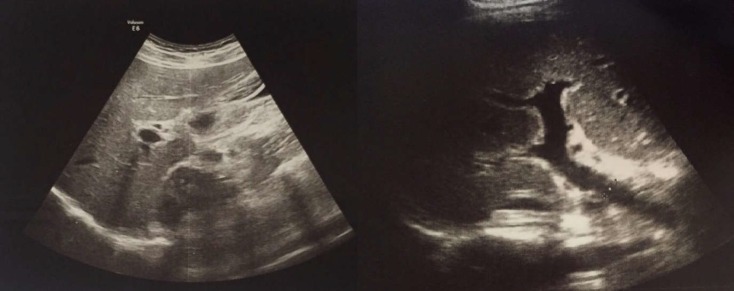
Sonography imaging of the liver

**Table 1:** Roussel Uclaf Causality Assessment Method (RUCAM) which suggests that glutamine consumption possibly induced hepatotoxicity

**Item**	**Score**
Are there previous conclusive reports on this reaction?	1
Did the adverse events appear after the suspected drug was given?	0
Did the adverse reaction improve when the drug was discontinued or a specific antagonist was given?	1
Did the adverse reaction appear when the drug was readministered?	0
Are there alternative causes that could have caused the reaction?	2
Did the reaction reappear when a placebo was given?	0
Was the drug detected in any body fluid in toxic concentrations?	0
Was the reaction more severe when the dose was increased, or less severe when the dose was decreased?	0
Did the patient have a similar reaction to the same or similar drugs in any previous exposure?	0
Was the adverse event confirmed by any objective evidence?	1
**Total Score**	**5**

Hemogram revealed thrombocytopenia [80,000 (150000-450000) per micro liter]. An evaluation was done for the causes of acute liver damage. They included viral hepatitis (hepatitis A, B, C, D, and E) and autoimmune hepatitis workup (antinuclear antibodies, antimitochondrial antibody, and immunoglobulin G). The results of the mentioned workups were negative. The anti–smooth muscle antibody (ASMA) was negative (less than 1/80). The result of herpes simplex virus (HSV) 1/2 IgG also was negative. The toxicology panel of the blood/urine of patient was also negative. This panel checked methadone, opium, tramadol, amphetamine, and tetrahydrocannabinol. The patient did not agree to have her liver examined via biopsy sampling. In addition, the sonography imaging of liver and abdomen showed that portal vein diameter was 10 mm and hepatoportal flow was 12 cm/s. The liver size was normal with a hyperechoic lesion (6 x 11 cm) in the right lobe and a contracted thickened gall bladder. Dynamic magnetic resonance imaging confirmed the lesion in the liver to be a hemangioma (Figure 2). It revealed a smooth, well-demarcated lesion with peripheral nodular enhancement in the arterial phase with progressive centripetal enhancement on delayed images, typical of hemangioma. Finally, diagnosis of glutamine-induced hepatotoxicity was supported based on the medical history, laboratory results, and imaging findings. In the presented case, multiple etiologies for the acute liver damage were excluded and causality assessment score (Score: 5) was calculated based on Roussel Uclaf Causality Assessment Method (RUCAM), which suggested that glutamine consumption possibly induced hepatotoxicity (Table 1) (8). The patient was admitted to the gastroenterology ward and glutamine consumption discontinued. Appropriate fluid therapy was started, and then pantoprazole tablet was given at a dose of 40 mg/day, Ursodeoxycholic acid capsule at a dose of 300 mg/TDS (9), and N-acetylcysteine tablet at a dose of 600 mg/BID (10). After two weeks of follow-up, the patient recovered uneventfully. The last visit of the patient was done after three months and she did not show any notable point and her medical condition was satisfying.

## Discussion

During intense physical activity, glutamine extraction increases by the liver. Consequently, gluconeogenesis or urea formation increases. Also, in this condition, kidneys and immune system cells use more glutamine (11, 12). Some studies also found that liver failure may be associated with a high plasma glutamine level (13). These mechanisms suggest that the glutamine metabolism may be conducted by the liver. Also, glutamine, zinc, carnitine, and benzoate sodium have been reported for the treatment of hepatic encephalopathy in cirrhotic patients (14, 15). Our patient had an irregular consumption of alcohol which might have potentiated glutamine toxicity. Based on the calculated RUCAM score and excluding other possible etiologies, the liver injury was found to be possibly due to glutamine consumption. To the best of our knowledge, this is the first report of glutamine-induced hepatotoxicity which may be directly related to glutamine. The liver injury noted in our patient might also be due to unknown adulterants or toxic as well as bacterial contaminants in such products which we could not fully assess, but warrants stringent pharmacovigilance and toxicology analysis in the future. Hence, it is essential to know the potential adverse effects of dietary supplements. Health care professionals especially physicians and pharmacists, as prescribers and dispensers of these products, must know that consumption of these products may be associated with serious side effects which could be directly related to the product components or due to adulteration of such products with toxic substances and contaminants. Also, the early symptoms of the adverse effects, for example abdominal pain in the case of hepatotoxicity, should be a reminder for athletes and coaches. Despite all of about this case, further investigation is required to find the real relationship between glutamine consumption and hepatotoxicity.

## Conflict of interests

The authors declare that they have no conflict of interest.

## References

[1] Ahmad J, Odin JA (2017). Epidemiology and Genetic Risk Factors of Drug Hepatotoxicity. Clin Liver Dis.

[2] Navarro V, Khan I, Björnsson E, Seeff LB, Serrano J, Hoofnagle JH (2017). Liver Injury from Herbal and Dietary Supplements. Hepatology.

[3] Knapik JJ, Steelman RA, Hoedebecke SS, Austin KG, Farina EK, Lieberman HR (2016). Prevalence of Dietary Supplement Use by Athletes: Systematic Review and Meta-Analysis. Sports Med.

[4] Thomas DT, Erdman KA, Burke LM (2016). Position of the Academy of Nutrition and Dietetics, Dietitians of Canada, and the American College of Sports Medicine: Nutrition and Athletic Performance. J Acad Nutr Diet.

[5] Suzic Lazic J, Dikic N, Radivojevic N, Mazic S, Radovanovic D, Mitrovic N (2011). Dietary supplements and medications in elite sport--polypharmacy or real need?. Scand J Med Sci Sports.

[6] Kamangar F, Emadi A (2012). Vitamin and Mineral Supplements: Do We Really Need Them?. Int J Prev Med.

[7] Mousavi S, Mansouri A, Jahangard-Rafsanjani Z, Sarayani A, Hadjibabaie M, Gholami K (2014). Bibliographic search of publication patterns in rational use of drugs in Iran: a systematic approach. Acta Medica Iranica.

[8] Danan G, Teschke R (2016). RUCAM in Drug and Herb Induced Liver Injury: The Update. Int J Mol Sci.

[9] Huang YS (2010). S1881 The Therapeutic Efficacy of Ursodeoxycholic Acid (UDCA) in Drug-Induced Liver Injury: Results of a Randomized Controlled Trial. Gastroenterology.

[10] Nabi T, Nabi S, Rafiq N, Shah A (2017). Role of N-acetylcysteine treatment in non-acetaminophen-induced acute liver failure: A prospective study. Saudi J Gastroenterol.

[11] Newsholme P, Procopio J, Lima MM, Pithon-Curi TC, Curi R (2003). Glutamine and glutamate--their central role in cell metabolism and function. Cell Biochem Funct.

[12] McCormack WP, Hoffman JR, Pruna GJ, Jajtner AR, Townsend JR, Stout JR (2015). Effects of l-Alanyl-l-Glutamine Ingestion on One-Hour Run Performance. J Am Coll Nutr.

[13] Helling G, Wahlin S, Smedberg M, Pettersson L, Tjader I, Norberg A (2016). Plasma Glutamine Concentrations in Liver Failure. PLoS One.

[14] Bismuth M, Funakoshi N, Cadranel JF, Blanc P (2011). Hepatic encephalopathy: from pathophysiology to therapeutic management. Eur J Gastroenterol Hepatol.

[15] Nazari MA, Malayeri SH, Pourhoseingholi MA, Mohebi SR, Zali MR (2010). Evaluation of zinc plasma level in Iranian cirrhotic patients due to hepatitis B and hepatitis C. Hepat Mon.

